# Production of Membrane Vesicles by *Enterococcus faecium* Cultured With or Without Subinhibitory Concentrations of Antibiotics and Their Pathological Effects on Epithelial Cells

**DOI:** 10.3389/fcimb.2019.00295

**Published:** 2019-08-14

**Authors:** Mi Hyun Kim, Se Yeon Kim, Joo Hee Son, Seung Il Kim, Hayoung Lee, Shukho Kim, Minsang Shin, Je Chul Lee

**Affiliations:** ^1^Department of Microbiology, School of Medicine, Kyungpook National University, Daegu, South Korea; ^2^Drug and Disease Target Team, Korea Basic Science Institute, Ochang, South Korea; ^3^Department of Bio-Analytical Science, University of Science and Technology (UST), Daejeon, South Korea

**Keywords:** *E. faecium*, antibiotics, membrane vesicle, cytotoxicity, inflammatory response

## Abstract

*Enterococcus faecium* is a clinically important pathogen associated with opportunistic infection and multi-drug resistance. *E. faecium* has been shown to produce membrane vesicles (MVs), but MV production by *E. faecium* under antibiotic stress conditions and the pathogenic traits thereof have yet to be determined. This study investigated the production of MVs in *E. faecium* ATCC 700221 cultured with sub-minimum inhibitory concentrations (MICs) of vancomycin or linezolid and determined their pathologic effects on colon epithelial Caco-2 cells. *E. faecium* ATCC 700221 cultured with 1/2 MIC of vancomycin or linezolid produced 3.0 and 1.5 times more MV proteins than bacteria cultured without antibiotics, respectively. Totals of 438, 461, and 513 proteins were identified in MVs from *E. faecium* cultured in brain heart infusion broth (MVs/BHI), BHI broth with 1/2 MIC of vancomycin (MVs/VAN), or BHI broth with 1/2 MIC of linezolid (MVs/LIN), respectively. Intact MVs/BHI induced cytotoxicity and the expression of pro-inflammatory cytokine and chemokine genes in Caco-2 cells in a dose-dependent manner, but proteinase K-treated MVs significantly suppressed these pro-inflammatory responses. MVs/LIN were more cytotoxic toward Caco-2 cells than MVs/BHI and MVs/VAN, whereas MVs/VAN stimulated more pro-inflammatory cytokine gene expression in Caco-2 cells than MVs/BHI and MVs/LIN. Overall results indicated that antibiotics modulate the biogenesis and proteomes of MVs in *E. faecium* at subinhibitory concentrations. MVs produced by *E. faecium* cultured under antibiotic stress conditions induce strong host cell responses that may contribute to the pathogenesis *E. faecium*.

## Introduction

Enterococci, gram-positive, facultative anaerobic bacteria, are among the most abundant commensal flora of the human and animal gut microbiomes (Shepard and Gilmore, 2002; Heidari et al., 2016). These microorganisms are of medical importance as opportunistic pathogens, especially in severely ill or immunocompromised hosts (Carmeli et al., 2002; da Silva et al., 2014). Of the medically important *Enterococcus* species, specifically *E. faecium* and *E. faecalis, E. faecium* causes emergent and challenging hospital-acquired infections due to its resistance to antibiotics (French, 1998; Shepard and Gilmore, 2002; Zirakzadeh and Patel, 2006; O'Driscoll and Crank, 2015; Heidari et al., 2017). *E. faecium* is listed among the “ESKAPE” pathogens that are potentially drug resistant and can cause life-threatening nosocomial infections (Rice, 2008; Pendleton et al., 2013). Colonization and infection of vancomycin-resistant enterococci (VRE) has dramatically increased over a short period of time in many countries (O'Driscoll and Crank, 2015). VRE has developed resistance to other classes of antibiotics, and VRE infections are more difficult to treat than other enterococcal infections because fewer therapeutic options, such as linezolid, daptomycin, and tigecycline, are available to kill these bacteria (Zirakzadeh and Patel, 2006; O'Driscoll and Crank, 2015). In addition to antimicrobial resistance, enterococci harbor several virulence factors, including major autolysin (AtlA), enterococcal leucine-rich repeat-containing protein (ElrA), enterococcal surface protein (Esp), cytolysin (CylA), and collagen-binding protein (Acm), which lead to them being opportunistic pathogens (Shankar et al., 2002; Eckert et al., 2006; Emirian et al., 2009; Arias and Murray, 2012; Dumoulin et al., 2013; Hendrickx et al., 2013; Heidari et al., 2016, 2017).

The production and secretion of outer membrane vesicles (OMVs) have been identified in many gram-negative pathogens (Kuehn and Kesty, 2005; Kulp and Kuehn, 2010). OMVs enhance bacterial pathogenesis by delivery of virulence factors to host cells, biofilm formation, increased antimicrobial resistance, and modulation of innate immune responses (Kuehn and Kesty, 2005; Kulp and Kuehn, 2010). Membrane vesicles (MVs) derived from gram-positive bacteria have been discovered in *Staphylococcus aureus, Bacillus* spp., *Clostridium* spp., and *Listeria monocytogenes* (Lee et al., 2009, 2013; Rivera et al., 2010; Gurung et al., 2011; Nicholas et al., 2017). MVs derived from gram-positive pathogens are potent facilitators of host-pathogen interactions via transport of virulence factors and modulation of immune response, similar to OMVs from gram-negative pathogens (Rivera et al., 2010; Gurung et al., 2011; Thay et al., 2013; Nicholas et al., 2017). The biogenesis of bacterial extracellular vesicles is still poorly understood, but stressful environments such as growth temperature, envelope stress, or oxidizing agents have been shown to increase their production (Mug-Opstelten and Witholt, 1978; Katsui et al., 1982; Thompson et al., 1985; McBroom and Kuehn, 2007). Subinhibitory concentrations of gentamicin and polymyxin B increase the production of OMVs in gram-negative pathogens (Kadurugamuwa and Beveridge, 1995; Macdonald and Kuehn, 2013). Moreover, treatment of gram-positive bacteria with β-lactam antibiotics stimulates the production of MVs through a weakening of the cell walls (Biagini et al., 2015; Toyofuku et al., 2017; Yun et al., 2018; Andreoni et al., 2019). Thus, the extracellular vesicles produced by pathogens under antibiotic stress conditions that are often encountered during infection may interact with host cells differently than those produced by pathogens under no antibiotic conditions.

Clinical *E. faecium* isolates have been shown to produce MVs during *in vitro* culture (Wagner et al., 2018). Several virulence factors and antimicrobial resistance-related proteins were identified in *E. faecium* MVs, suggesting that *E. faecium* MVs may augment pathogenesis and antimicrobial resistance. However, the pathogenic attributes of *E. faecium* MVs toward host cells have yet to be determined. The aim of this study was to explore the production of MVs in *E. faecium* cultured with subinhibitory concentrations of different antibiotics and investigate the ability of these MVs to induce cytotoxicity and pro-inflammatory responses in colon epithelial cells.

## Materials and Methods

### Bacterial Strains

*E. faecium* ATCC 700221 obtained from the American Type Culture Collection (Manassas, VA, USA) was used in this study. Bacteria were grown in brain heart infusion (BHI) media (BD Biosciences, San Jose, CA, USA) at 37°C.

### Cell Culture

Caco-2 cells originating from a heterogeneous colorectal adenocarcinoma were purchased from the Korean Cell Line Bank (Seoul, Korea). Cells were grown in minimal essential medium (HyClone, Logan, UT, USA) supplemented with 10% fetal bovine serum (HyClone), 2 mM l-glutamine, 1,000 U/ml penicillin G, and 50 μg/ml streptomycin at 37°C in a humidified atmosphere with 5% CO_2_.

### Antimicrobial Susceptibility Test

The minimum inhibitory concentrations (MICs) of vancomycin and linezolid were determined by microdilution methods according to the Clinical Laboratory Standards Institute (Clinical Laboratory Standards Institute, 2015). *E. faecalis* ATCC 29212 and *S. aureus* ATCC 29213 were used as quality control strains.

### Isolation of MVs

MVs produced by *E. faecium* ATCC 700221 were isolated from bacterial culture supernatants as previously described (Gurung et al., 2011; Kim et al., 2016; Yun et al., 2018). *E. faecium* was cultured in 500 ml of BHI broth at 37°C with shaking. Bacteria were cultured in 500 ml of BHI broth supplemented with 1/2 MIC of vancomycin (256 μg/ml) or linezolid (1 μg/ml) at 37°C with shaking to isolate MVs from *E. faecium* under antibiotic stress conditions. Bacteria were cultured to late exponential phase (Figure S1). Bacterial cells were centrifuged at 6,000g for 15 min at 4°C. The culture supernatants were filtered using a QuixStand Benchtop System (GE Healthcare, Amersham, UK) with a 0.2 μm hollow fiber membrane (GE Healthcare), and concentrated using a QuixStand Benchtop System with a 500 kDa hollow fiber membrane (GE Healthcare). MV samples were ultracentrifuged at 150,000g for 3 h at 4°C and washed in phosphate-buffered saline (PBS) followed by another ultracentrifugation cycle, after which MV pellets were resuspended in PBS. The protein concentrations of the MVs were measured using a modified BCA assay (Thermo Scientific, Waltham, MA, USA). Purified MVs were verified the sterility by streaking on blood agar plates and then stored at −80°C until use. *E. faecium* MVs were treated with proteinase K (0.1 μg/ml) (Biofact, Daejeon, Korea) for 3 h at 50°C for the degradation of MV proteins (Jeon et al., 2016).

### Sodium Dodecyl Sulfate-Polyacrylamide Gel Electrophoresis (SDS-Page)

Bacteria were cultured to reach late exponential phase and then disrupted by sonication (Branson Ultrasonics Corp., Danbury, CT, USA). Proteins in the culture supernatant were precipitated with 10% trichloroacetic acid. The bacterial lysate, culture supernatants, and MVs corresponding to 10 μg of protein were mixed with SDS-PAGE sample buffer (1 M Tris HCl [pH 6.8], 10% SDS, 1% bromophenol blue, glycerol, and β-mercaptoethanol) and boiled for 10 min. The samples were then separated on a 10% SDS-PAGE gel, and gels were stained with Coomassie brilliant blue R-250 (Bio-Rad, Hercules, CA, USA).

### Transmission Electron Microscopic (TEM) Analysis

Purified *E. faecium* MVs samples were applied onto copper grids and stained with 1 or 2% uranyl acetate. Samples were visualized using a transmission electron microscope (HT-7700, Hitachi, Japan) operating at 120 kV.

### Identification of Proteins in *E. faecium* MVs

Proteins in the MVs of *E. faecium* ATCC 700221 were identified using one-dimensional gel electrophoresis and liquid chromatography-tandem mass spectrometry (1-DE-LC-MS/MS) as previously described (Yun et al., 2018). Fifteen μg of MV proteins were separated on a 12% SDS-PAGE. The gel was fractionated eight fractions according to molecular weight. Tryptic in-gel digestion was performed as previously described (Choi et al., 2014). Digested peptides were dissolved in sample buffer (0.02% acetic acid and 0.1% formic acid). Peptides were loaded onto a 2G-V/V trap column (Waters, Milford, MA, USA). Peptides were directed onto a 10 cm × 75 μm (i.d.) C18 reversed-phase column at a flow rate of 300 nL/min. High performance liquid chromatography conditions and search parameters for tandem mass spectrometry (MS/MS) analysis were applied as described previously (Choi et al., 2014). All MS and MS/MS spectra obtained using the LTQ-Velos ESI ion trap mass spectrometer were acquired in the data-dependent mode. Nano-LC-MS/MS spectra were searched with MASCOT version 2.4 (Matrix Science, UK) using protein sequences from the genome of *E. faecium* ATCC 700221 as a reference. A proteomic analysis was performed three times for each MV sample. Only proteins identified in three experiments were analyzed. The locations of MV proteins were predicted using the subcellular location prediction program, CELLO version 2.5 (http://cello.life.nctu.edu.tw/), per reference locations in UniProt (https://www.uniprot.org/). Proteins in *E. faecium* MVs were classified based on Gene Ontology (GO) functions using the Blast2GO 5.2 program (https://www.blast2go.com/).

### Cell Viability Tests

The viability of Caco-2 cells was measured using the 3-[4,5-dimethylthiazol-2-yl]-2,5 diphenyltetrazolium bromide (MTT) assay (Abcam, Cambridge, UK). Cells were seeded at a concentration of 1.0 × 10^5^/well in a 24-well microplate. Caco-2 cells were treated with *E. faecium* MVs for 24 h and then cell growth was measured at 570 nm, after 2 h of incubation with the MTT reagent.

### Flow Cytometric Analyses

Caco-2 cells were seeded at a concentration of 2.5 × 10^5^ cells/ml in 6-well plates. After treatment with 20 μg/ml of *E. faecium* MVs for 24 h, cells were stained with fluorescein isothiocyanate (FITC)-conjugated annexin V and propidium iodide (PI) (BD Biosciences, San Jose, CA, USA). The samples were analyzed in a FACSCalibur flow cytometer (BD Biosciences) by plotting PI and annexin V fluorescent intensities. For each sample, 10,000 cells were acquired for data analysis.

### Quantitative Real-Time Polymerase Chain Reaction (qPCR) of Pro-inflammatory Cytokine and Chemokine Genes

The expression of genes encoding glyceraldehyde 3-phosphate dehydrogenase (GAPDH), interleukin (IL)-1β, IL-6, IL-8, tumor necrosis factor (TNF)-α, and monocyte chemoattractant protein (MCP)-1 was assessed by qPCR as described previously (Jun et al., 2013; Nho et al., 2015). Caco-2 cells were seeded in 6-well plates and then treated with 1, 2, or 5 μg/ml of *E. faecium* MVs for 3 h. Total RNA was extracted using an RNeasy Mini Kit (Qiagen, Valencia, CA, USA). cDNA was synthesized by reverse transcription of 1 μg of total RNA using oligo dT primers and M-MLV reverse transcriptase (Enzynomics, Daejeon, Korea). Gene expression was quantified using TOPreal™ qPCR 2X PreMIX (SYBR Green with high ROX) (Enzynomics) with an ABI PRISM 7500 Real-Time System (Applied Biosystems, Foster city, CA, USA). Fold changes in gene expression were calculated using the comparative Ct method. The expression levels of genes were normalized to GAPDH expression levels. Each experiment was performed in triplicate.

### Statistical Analysis

Data were analyzed using R 3.3.4 (https://www.r-project.org/). The expression of genes was analyzed using one-way ANOVA with Dunnett's *post-hoc* test. Cell death and cytokine gene expression between intact MVs and proteinase K-treated MVs were analyzed using Student's *t*-test. Differences of *P* < 0.05 were considered as statistically significant.

## Results

### MV Production in *E. faecium* ATCC 700221 Cultured With or Without Antibiotics

**Figure 1:**
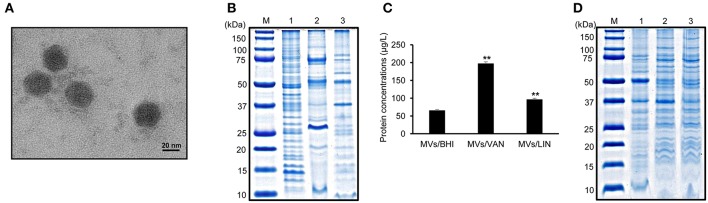
Membrane vesicles (MVs) produced by *E. faecium* ATCC 700221 cultured with or without antibiotics. **(A)** Transmission electron micrographs of MVs from *E. faecium* cultured in BHI broth. **(B)** SDS-PAGE analysis of proteins obtained from bacterial lysates (lane 1), culture supernatant (lane 2), and MVs (lane 3). Bacteria were cultured in BHI broth to late exponential phase. Lane M, molecular weight marker. **(C)** Production of MVs from *E. faecium* cultured with or without antibiotics. MVs were isolated from *E. faecium* cultured in BHI broth (MVs/BHI), BHI broth with 256 μg/ml vancomycin (MVs/VAN), or BHI broth with 1 μg/ml linezolid (MVs/LIN). The protein concentration of MVs isolated from 1 L of bacterial culture was measured using a modified BCA assay. Data are presented as the mean ± SD of three independent experiments. ^**^P < 0.01 compared to MVs/BHI. **(D)** SDS-PAGE analysis of MV proteins. Lane M, molecular weight marker; 1, MVs/BHI; 2, MVs/VAN; 3, MVs/LIN. **(A,B,D)** represent one of three independent experiments.

To determine whether *E. faecium* ATCC 700221 produced MVs, bacteria were cultured in BHI broth to late exponential phase (Figure S1) and MVs were isolated from the culture supernatant. TEM analysis showed that *E. faecium* produced spherical MVs sized ≤50 nm (Figure 1A). SDS-PAGE analysis showed the different protein profiles among bacterial lysates, culture supernatant, and MVs (Figure 1B). *E. faecium* ATCC 700221 were resistant to vancomycin (MIC 512 μg/ml), but susceptible to linezolid (MIC 2 μg/ml). *E. faecium* were cultured in BHI broth with 1/2 MIC of vancomycin (256 μg/ml) or linezolid (1 μg/ml), and MVs were isolated from the culture supernatants to determine the effect of subinhibitory concentrations of antibiotics on MV production and their protein profiles. *E. faecium* cultured with 1/2 MIC of vancomycin or linezolid produced 3.0 (197.0 ± 5.4 μg/L) and 1.5 (95.8 ± 3.9 μg/L) times more MV proteins than bacteria cultured in BHI broth without antibiotics (65.0 ± 3.2 μg/L), respectively (Figure 1C). Moreover, SDS-PAGE analysis showed the different protein profiles among MVs of *E. faecium* cultured in BHI broth without antibiotics (MVs/BHI), with vancomycin (MVs/VAN), or with linezolid (MVs/LIN) (Figure 1D). These results suggest that antibiotics modulate MV biogenesis of *E. faecium* ATCC 700221 at subinhibitory concentrations.

### Proteomes of MVs of *E. faecium* ATCC 700221 Cultured With or Without Antibiotics

**Figure 2:**
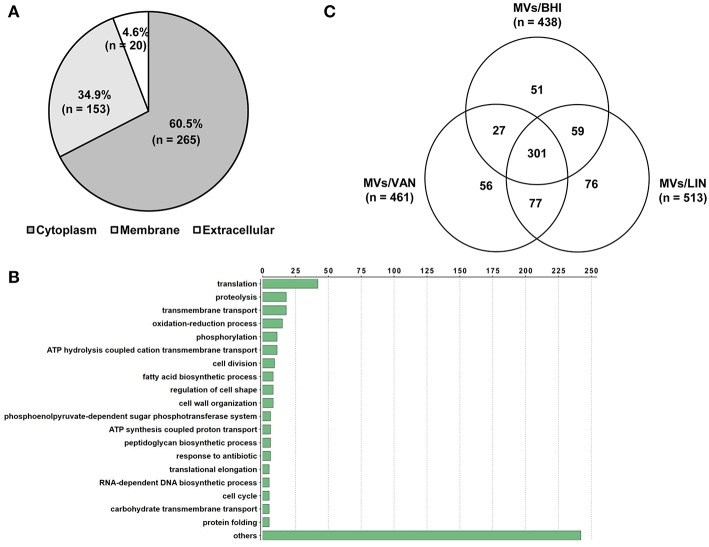
Proteomic analysis of MVs produced by *E. faecium* ATCC 700221. MVs were isolated from *E. faecium* cultured in BHI broth (MVs/BHI), BHI broth with 256 μg/ml vancomycin (MVs/VAN), or BHI broth with 1 μg/ml linezolid (MVs/LIN). **(A,B)** A total of 438 proteins in the MVs/BHI were analyzed based on cellular localization **(A)** and Gene Ontology **(B)**. **(C)** A Venn diagram of proteins identified in the MVs/BHI, MVs/VAN, and MVs/LIN.

A proteomic analysis was performed to identify proteins in *E. faecium* MVs. A total of 438 proteins were identified in MVs/BHI (Table S1). Of these 438 proteins, 265, 153, and 20 were identified to be located in the cytoplasm, membrane, and extracellular compartments, respectively (Figure 2A). A total of 371 proteins in the MVs/BHI were classified by GO functions, but 67 proteins were not classified into any GO group. Translation-associated proteins (*n* = 42) were the most common (Figure 2B). Next, to determine the effect of antibiotics on the protein profiles of *E. faecium* MVs, the proteomes of MVs/LIN and MVs/VAN were analyzed. A total of 461 and 513 proteins were identified in MVs/VAN and MVs/LIN, respectively (Table S1). A total of 301 proteins were commonly identified in all three *E. faecium* MVs, whereas 51, 56, and 76 proteins were specifically identified in MVs/BHI, MVs/VAN, and MVs/LIN, respectively (Figure 2C). Cellular localization of MV proteins was similar among MVs/BHI, MVs/LIN, and MVs/VAN (Figure S2), but the prevalence of MV proteins belonging to GO functional groups was different for the three *E. faecium* MVs (Figure S3).

*E. faecium* ATCC 700221 harbors a plasmid-mediated *vanA* cluster that is responsible for resistance to vancomycin. The proteins encoded by the *vanA* cluster, including VanA, VanH, VanR, VanS, VanX, and VanZ, and aminoglycoside-modifying enzyme AphA were identified in MVs/VAN (Table 1). Cadmium resistance protein CadD and tellurite resistance protein TelA were also identified in MVs/VAN. Putative virulence factors, including autolysin (AMQ96750.1), collagen-binding protein (AMQ98256.1), catabolite control protein A (AMQ97727.1), fibronectin-binding protein (AMQ97173.1), and pilus assembly protein (AMQ98642.1), were also associated with *E. faecium* MVs. However, the relative amounts of antimicrobial resistance- and virulence-associated proteins in *E. faecium* MVs varied according to bacterial culture conditions (Table 1 and Table S1). These results suggest that subinhibitory concentrations of antibiotics modulate the proteomes of *E. faecium* MVs.

**Table 1:** Identification of antimicrobial resistance-and virulence-associated proteins in *E. faecium* MVs.

**Protein**	**Functional description**	**Protein ID**	**MVs/BHI**	**MVs/VAN**	**MVs/LIN**
VanA	D-alanyl-D-alanine dipeptidase	AMQ98821.1	O^*^	O	O
VanH	Alpha-keto acid dehydrogenase	AMQ98822.1	X	O	X
VanR	PhoB family transcriptional regulator	AMQ98437.1	X	O	O
VanS	Histidine kinase	AMQ98824.1	O	O	O
VanX	Peptidase M15	AMQ98820.1	O	O	X
VanZ	Protein vanZ	AMQ98819.1	X	O	O
AphA	Aminoglycoside phosphotransferase	AMQ98790.1	X	O	O
EmrB	Multidrug transporter	AMQ96422.1	X	O	X
DhaS	TetR family transcriptional regulator	AMQ96225.1	X	O	O
ErmB	SAM-dependent methyltransferase	AMQ98797.1	O	O	O
CadD	Cadmium resistance protein	AMQ98688.1	X	O	X
TelA	Tellurite resistance protein	AMQ96515.1	O	O	O
ArpU	Autolysin	AMQ96750.1	O	X	X
SdrD	Collagen-binding protein	AMQ98256.1	O	X	O
CcpA	Catabolite control protein A	AMQ97727.1	O	O	O
PavA	Fibronectin-binding protein	AMQ97173.1	X	X	O
-	Pilus assembly protein	AMQ98642.1	O	X	O

* *O, presence of proteins; X, absence of proteins*.

### Host Cell Cytotoxicity Against MVs of *E. faecium* Cultured With or Without Antibiotics

**Figure 3:**
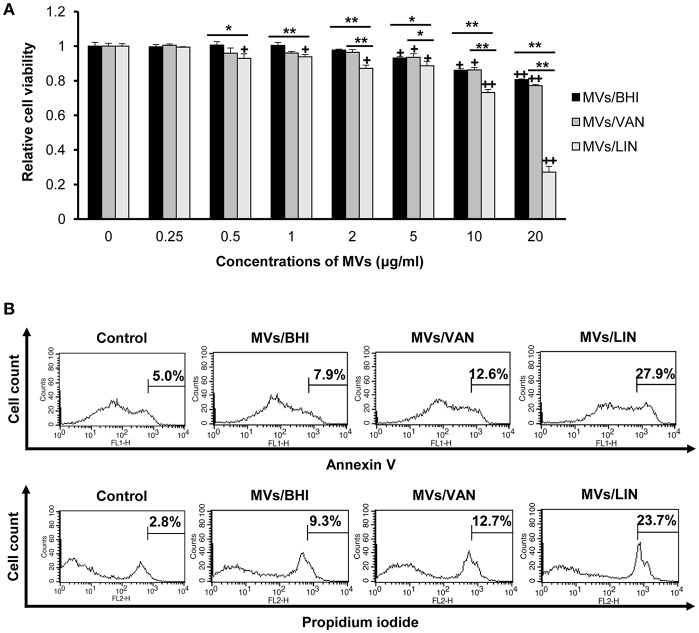
Cytotoxicity of Caco-2 cells treated with MVs from *E. faecium* ATCC 700221. MVs were isolated from culture supernatants of *E. faecium* cultured in BHI broth (MVs/BHI), BHI broth with 256 μg/ml vancomycin (MVs/VAN), or BHI broth with 1 μg/ml linezolid (MVs/LIN). Cells were treated with various concentrations of *E. faecium* MVs for 24 h. **(A)** Cell viability was determined using an MTT assay. Data are presented as the mean ± SD of three independent experiments. ^+^*P* < 0.05, ^++^*P* < 0.01 compared to untreated control cells. *P < 0.05, **P < 0.01 among the same concentrations of MVs/BHI, MVs/VAN, or MVs/LIN. **(B)** Flow cytometric analysis of Caco-2 cell death induced by *E. faecium* MVs. Cells were treated with 20 μg/ml of *E. faecium* MVs for 24 h. Cells were stained with FITC-Annexin V and propidium iodide, and 10^4^ cells were counted. The figure represents one of three independent experiments that yielded similar results.

To determine whether MVs produced by *E. faecium* ATCC 700221 could induce cytotoxicity in human colon epithelial cells, Caco-2 cells were treated with *E. faecium* MVs (0.25–20 μg/ml protein) for 24 h, and the viability of cells was analyzed using an MTT assay. Cytotoxicity was observed in Caco-2 cells treated with ≥5 μg/ml of MVs/BHI and MVs/VAN and in Caco-2 cells treated with ≥0.5 μg/ml of MVs/LIN (Figure 3A). Cytotoxicity was significantly different between MVs/BHI and MVs/LIN at ≥0.5 μg/ml, and between MVs/VAN and MVs/LIN at ≥2 μg/ml. Live bacteria at multiplicity of infection (MOI) 10 were more cytotoxic to Caco-2 cells than 5 μg/ml of MVs/BHI, but cytotoxic activity was similar between dead bacteria at MOI 100 and 5 μg/ml of MVs/BHI (Figure S4). When Caco-2 cells were treated with 256 μg/ml of vancomycin or 1 μg/ml of linezolid as a negative control, no cytotoxicity was observed. Cell death induced by *E. faecium* MVs was further analyzed with flow cytometry wherein Caco-2 cells were treated with 20 μg/ml of MVs/BHI, MVs/VAN, or MVs/LIN for 24 h. MVs/LIN were more cytotoxic to Caco-2 cells than MVs/BHI or MVs/VAN (Figure 3B). These results suggest that *E. faecium* MVs contain factors that are cytotoxic to host cells, and that *E. faecium* exposed to subinhibitory concentrations of linezolid produces more cytotoxic MVs than under exposure to subinhibitory concentrations of vancomycin or no antibiotics.

### Pro-inflammatory Responses in Caco-2 Cells Treated With MVs of *E. faecium* Cultured With or Without Antibiotics

**Figure 4:**
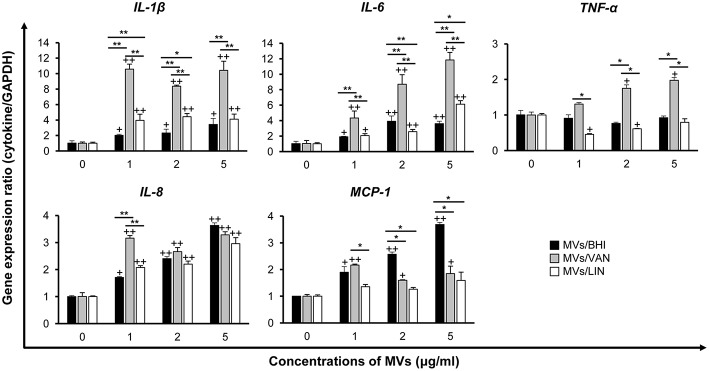
Expression of pro-inflammatory cytokine and chemokine genes in Caco-2 cells treated with MVs from *E. faecium* ATCC 700221. MVs were isolated from culture supernatants of *E. faecium* cultured in BHI broth (MVs/BHI), BHI broth with 256 μg/ml vancomycin (MVs/VAN), or BHI broth with 1 μg/ml linezolid (MVs/LIN). Cells were treated with various concentrations of *E. faecium* MVs for 3 h and gene expression was assessed via qPCR. Data are presented as the mean ± SD of three independent experiments. ^+^*P* < 0.05, ^++^*P* < 0.01 compared to untreated control cells. *P < 0.05, **P < 0.01 among the same concentrations of MVs/BHI, MVs/VAN, or MVs/LIN.

To determine whether *E. faecium* MVs could induce pro-inflammatory responses, Caco-2 cells were treated with 1, 2, or 5 μg/ml of MVs/BHI, MVs/VAN, or MVs/LIN for 3 h, and the expression of genes encoding pro-inflammatory cytokines such as IL-1β, IL-6, and TNF-α, and chemokines such as IL-8 and MCP-1 was analyzed using qPCR. Cytotoxicity was not observed in Caco-2 cells treated with ≤5 μg/ml of *E. faecium* MVs for 3 h (data not shown). The expression of *IL-1*β, *IL-6, IL-8*, and *MCP-1* genes significantly increased in cells treated with ≥1 μg/ml of MVs/BHI (Figure 4). However, MVs/BHI did not stimulate the expression of *TNF*-α in cells treated with ≤5 μg/ml. MVs/VAN stimulated the expression of all cytokine genes tested in cells treated with ≥1 μg/ml. MVs/LIN stimulated the expression of *IL-1*β, *IL-6*, and *IL-8* at ≤5 μg/ml, whereas MVs/LIN significantly down-regulated the expression of *TNF*-α in cells treated with 1 and 2 μg/ml of MVs/LIN. The expression levels of *IL-1*β, *IL-6*, and *TNF*-α genes were significantly increased in cells treated with MVs/VAN compared to levels in cells treated with MVs/BHI or MVs/LIN. However, MVs/VAN did not significantly increase the expression of *IL-8* and *MCP-1* genes in Caco-2 cells as compared to MVs/BHI and MVs/LIN, with the exception of *IL-8* and *MCP-1* in cells treated with 1 μg/ml of MVs/VAN. MVs/BHI (5 μg/ml) induced more *IL-8* gene expression than dead bacteria at MOI 10 and 100 and live bacteria at MOI 10 (Figure S5). Vancomycin (256 μg/ml) and linezolid (1 μg/ml) did not induce the expression of *IL-8* gene in Caco-2 cells. These results suggest that MVs/VAN induce stronger pro-inflammatory responses in Caco-2 cells than MVs/BHI and MVs/LIN.

### Suppression of Pro-Inflammatory Responses to Proteinase K-Treated *E. faecium* MVs

**Figure 5:**
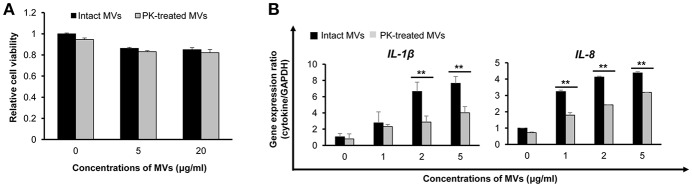
Host cell responses to proteinase K (PK)-treated *E. faecium* MVs. **(A)** Caco-2 cells were incubated with intact or PK-treated MVs/BHI for 24 h and cell viability was determined using an MTT assay. Data are presented as the mean ± SD of three independent experiments. **(B)** Cells were incubated with intact or PK-treated MVs/BHI for 3 h and gene expression was assessed via qPCR. Data are presented as the mean ± SD of three independent experiments. **P < 0.01 comparing intact and PK-treated MVs.

MVs isolated from *E. faecium* ATCC 700221 cultured under different antibiotic stress conditions exhibited different proteomes and host cell responses. To determine whether *E. faecium* MV proteins contributed to cytotoxicity and pro-inflammatory responses in host cells, MVs/BHI were treated with proteinase K and MV proteins were degraded. Most MV proteins were degraded, but some MV proteins were resistant to proteinase K (Figure S6). Caco-2 cells were incubated with either intact or proteinase K-treated MVs for 24 h. Cell death was not significantly different between intact MVs and proteinase K-treated MVs (Figure 5A). These results suggest that non-protein molecules or proteinase K-resistant proteins in *E. faecium* MVs contributes to host cell cytotoxicity. Next, Caco-2 cells were incubated with intact or proteinase K-treated MVs for 3 h and the expression levels of two selected cytokine genes, *IL-1*β and *IL-8*, were analyzed using qPCR. Even though there were some differences in the expression levels of *IL-1*β between MV batches (as shown in Figures 4, 5B), the expression of *IL-1*β and *IL-8* was significantly decreased in cells incubated with proteinase K-treated MVs compared to that in cells treated with intact MVs (Figure 5B). These results suggest that proteinase K-degradable MV proteins contribute to pro-inflammatory responses in Caco-2 cells.

## Discussion

Antibiotics modulate bacterial physiology regarding bacterial growth, gene transcription, and toxin production at subinhibitory concentrations (Goh et al., 2002; Yim et al., 2011; Moura et al., 2015; Ramos et al., 2015; Sinel et al., 2017). This study demonstrates that antibiotics at subinhibitory concentrations can modulate the biogenesis and proteomes of MVs in *E. faecium*. Moreover, MVs produced by *E. faecium* cultured under different antibiotic stress conditions also induce different levels of cytotoxicity and pro-inflammatory responses in colon epithelial cells *in vitro*.

The production of MVs in *E. faecium* was first described in clinical isolates (Wagner et al., 2018). Four *E. faecium* strains produced spherical-shaped MVs, but MV size varied among strains. MVs derived from three of the *E. faecium* strains were similar in size at ≤50 nm in diameter, whereas MVs of *E. faecium* K59-68 were measured at an average of 83 nm in diameter. Proteins identified in *E. faecium* MVs also varied among strains and bacterial growth phases. MVs of *E. faecium* grown to stationary phase contained more diverse proteins than MVs of *E. faecium* grown to exponential phase. The number of proteins identified in the MVs of four clinical *E. faecium* strains cultured in BHI broth to exponential phase ranged from 162 to 605. In the present study, *E. faecium* ATCC 700221 produced spherical-shaped MVs with a size ≤50 nm. Different protein profiles were observed among bacterial lysates, culture supernatants, and MVs. A total of 438 proteins were identified in MVs/BHI of *E. faecium* ATCC 700221. Moreover, cytotoxicity and *IL-8* gene expression in host cells were different between dead bacteria and *E. faecium* MVs. These results suggest that *E. faecium* MVs are not dead bacterial cells or bacterial debris, but a specific nanocomplex that carries specific proteins and other molecules derived from bacteria. Furthermore, MV size and proteome differ among *E. faecium* strains.

MVs isolated from *E. faecium* cultured under antibiotic stress conditions contained a more diverse array of proteins than MVs from *E. faecium* cultured without antibiotics. Moreover, *E. faecium* produced more MVs under vancomycin stress condition than under linezolid stress condition, whereas the number of MV proteins was more diverse under linezolid stress condition than under vancomycin stress condition. Reductions in peptidoglycan cross-linking have been shown to increase MV production in *S. aureus* (Wang et al., 2018). Vancomycin inhibits cross-linking of *N*-acetylmuramic acid with *N*-acetylglucosamine. Subinhibitory concentrations of vancomycin may thus result in a loosening of peptidoglycan layers leading to increased MV production in *E. faecium*. *E. faecium* MVs contained a diverse array of proteins that localized to the membrane, cytosol, and extracellular compartments, with a high abundance of ribosomal proteins. This recapitulates data from previous studies on MVs derived from other gram-positive bacteria (Lee et al., 2009, 2013; Gurung et al., 2011; Nicholas et al., 2017). However, the composition and relative amounts of proteins varied among MVs/BHI, MVs/VAN, and MVs/LIN. While linezolid blocks the initiation of protein synthesis, the number of proteins in MVs/LIN was the highest of all three *E. faecium* MVs. Although both vancomycin and linezolid are bactericidal toward *E. faecium*, why they modulate bacterial transcription and translation profiles in different ways remains to be investigated. Virulence factors such as collagen-binding protein (SdrD), CcpA, fibronectin-binding protein (PavA), and pilus assembly protein were associated with MVs/LIN, whereas proteins of the *vanA* cluster, the aminoglycoside-modifying enzyme AphA, the ribosome methylase ErmB, and transport proteins were found in MVs/VAN. In a previous study, the virulence factors Acm, CapD, Fnm, PilA2, PrpA, PtsD, and Scm, associated with bacterial adherence and colonization, AtlA, Esp, and SagA associated with biofilm formation, and CcpA associated with bacterial growth were identified in *E. faecium* MVs, but their associations therein differed among bacterial strains and growth phases (Wagner et al., 2018). The present study identified autolysin ArpU, CcpA, SdrD, PavA, and pilus assembly protein in *E. faecium* ATCC 700221 MVs. However, their association with MVs varied among MVs/BHI, MVs/LIN, and MVs/VAN. Antibiotics can activate or repress gene transcription and modulate MV biogenesis in bacteria at subinhibitory concentrations. This phenomenon may result in differences in protein components, including virulence factors, and antimicrobial resistance-associated proteins, between MVs/VAN and MVs/LIN.

*E. faecium* MVs induced host cell cytotoxicity in a dose-dependent manner. MVs/LIN showed the highest cytotoxic activity toward Caco-2 cells among the three *E. faecium* MVs. Lipoteichoic acid, extracellular superoxide anion, gelatinase, hyaluronidase, and cytolysin in *E. faecalis* or *E. faecium* contribute to tissue damage in the hosts (Kayaoglu and Ørstavik, 2004; Fisher and Phillips, 2009; Arias and Murray, 2012). However, these cytotoxic factors were not identified in *E. faecium* MVs in the present study. Moreover, no significant differences in host cell cytotoxicity were shown between intact and proteinase K-treated MVs, suggesting that non-protein components and/or proteinase K-resistant proteins in *E. faecium* MVs are responsible for this cytotoxicity, even though our previous study demonstrated that proteins associated with *S. aureus* MVs played a role in host cell cytotoxicity (Jeon et al., 2016). Further, α-hemolysin associated with *S. aureus* MVs was found to contribute to host cell cytotoxicity (Thay et al., 2013). Although the specific cytotoxic factors associated with *E. faecium* MVs were not determined in the present study, MVs produced by *E. faecium* cultured with subinhibitory concentrations of linezolid were highly cytotoxic.

MVs derived from gram-positive bacteria contain many pathogen-associated molecular patterns (PAMPs), such as membrane and cytoplasmic proteins, flagellins, peptidoglycans, and DNA (Lee et al., 2009; Jun et al., 2017; Liu et al., 2018). These bacterial ligands in the MVs may interact with specific receptors on host cells and thereby induce inflammatory responses. Our previous study showed that *S. aureus* MVs stimulated the expression of *IL-1*β, *IL-6, IL-8*, and *MIP-1*α genes in keratinocytes (Jun et al., 2017). Nucleotide-binding oligomerization domain 2 and Toll-like receptor 2 have been associated with peptidoglycans in *S. aureus* MVs and are implicated in the signaling pathways of inflammatory responses (Jun et al., 2017). In the present study, *E. faecium* MVs stimulated the expression of pro-inflammatory cytokine genes *IL-1*β and *IL-6* and chemokine genes *IL-8* and *MCP-1* in Caco-2 cells similarly to *S. aureus* MVs. However, proteinase K-treated *E. faecium* MVs significantly suppressed the expression of these cytokine genes as compared to intact MVs, suggesting that protein components in *E. faecium* MVs are largely responsible for pro-inflammatory responses in Caco-2 cells, unlike *S. aureus* MVs. An alternative explanation is that the degradation of surface proteins by proteinase K could have indirectly modulated other surface components. Because proteinase K-treated *E. faecium* MVs did not completely suppress the expression of these cytokine genes, it is possible that non-protein components such as peptidoglycans or proteinase K-resistant proteins of *E. faecium* MVs are also associated with pro-inflammatory responses in the host cells.

The expression of pro-inflammatory cytokine genes *IL-1*β, *IL-6*, and *TNF*-α was at the highest level in cells treated with MVs/VAN, whereas the expression of *MCP-1* gene was at the highest level in cells treated with MVs/BHI. These results suggest that antibiotics modulate the association of PAMPs with *E. faecium* MVs, which, in turn, influence pro-inflammatory responses in host cells. Further studies are required to identify specific PAMPs in *E. faecium* MVs and elucidate the signaling pathways associated with these pro-inflammatory responses.

Antibiotics affected the clinical outcomes of patients with *E. faecalis* bacteremia (Foo et al., 2014). Of the patients who treated with β-lactams or glycopeptides, glycopeptide therapy increased mortality of the patients with *E. faecalis* bacteremia (Foo et al., 2014). Several reports also demonstrated that glycopeptides rather than β-lactams were associated with increased patient treatment failure and mortality (Kim et al., 2008; Schweizer et al., 2011). Moreover, subinhibitory concentrations of antibiotics increase biofilm formation in multiple bacterial species, including *E. faecalis* (Kaplan, 2011; Andersson and Hughes, 2014; Yu et al., 2017). In the present study, subinhibitory concentrations of vancomycin enhanced the production of MVs in *E. faecium*. MVs produced by *E. faecium* cultured with subinhibitory concentrations of vancomycin induced stronger pro-inflammatory responses than MVs produced by *E. faecium* with subinhibitory concentrations of linezolid *in vitro*. The possible association of *E. faecium* MVs with bacterial pathogenesis and the clinical courses of infected patients treated with different antibiotics should be studied in greater depth in the future.

This study demonstrates that subinhibitory concentrations of antibiotics modulate MV-mediated host cell pathology in ways that are distinct from their antibacterial effects. The production of MVs in *E. faecium* under antibiotic stress conditions and the pathologic effects thereof on host cells help improve our understanding of the functional importance of bacterial extracellular vesicles.

## Data Availability

The raw data supporting the conclusions of this manuscript will be made available by the authors, without undue reservation, to any qualified researcher.

## Author Contributions

MK, SYK, and JS performed the isolation of MVs from bacteria cultured with or without antibiotic stress conditions. MK and SYK performed the cytotoxicity, the expression of cytokine genes and performed TEM analysis. MK, SIK, and HL performed proteomic analysis of MVs. SK, MS, and JL analyzed the data obtained. All authors contributed to the design of the experiments, analysis of the data, and writing and revision of the manuscript.

### Conflict of Interest Statement

The authors declare that the research was conducted in the absence of any commercial or financial relationships that could be construed as a potential conflict of interest.

## References

[B1] Andersson D. I. Hughes D. (2014). Microbiological effects of sublethal levels of antibiotics. Nat. Rev. Microbiol. 12, 465–478. 10.1038/nrmicro3270 24861036

[B2] Andreoni F. Toyofuku M. Menzi C. Kalawong R. Shambat S. M. François P. (2019). Antibiotics stimulate vesicles formation in *Staphylococcus aureus* in a phage-dependent and independent fashion and via different routes. Antimicrob. Agents Chemother. 63, e01439–e01418. 10.1128/AAC.01439-18 30509943 PMC6355553

[B3] Arias C. A. Murray B. E. (2012). The rise of the *Enterococcus*: beyond vancomycin resistance. Nat. Rev. Microbiol. 10, 266–278. 10.1038/nrmicro2761 22421879 PMC3621121

[B4] Biagini M. Garibaldi M. Aprea S. Pezzicoli A. Doro F. Becherelli M.. (2015). The human pathogen *Streptococcus pyogenes* releases lipoproteins as lipoprotein-rich membrane vesicles. Mol. Cell. Proteomics 14, 2138–2149. 10.1074/mcp.M114.045880 26018414 PMC4528243

[B5] Carmeli Y. Eliopoulos G. Mozaffari E. Samore M. (2002). Health and economic outcomes of vancomycin-resistant enterococci. Arch. Intern. Med. 162, 2223–2228. 10.1001/archinte.162.19.2223 12390066

[B6] Choi C. W. Park E. C. Yun S. H. Lee S. Y. Lee Y. G. Hong Y.. (2014). Proteomic characterization of the outer membrane vesicle of *Pseudomonas putida* KT2440. J. Proteome Res. 13, 4298–4309. 10.1021/pr500411d 25198519

[B7] Clinical and Laboratory Standards Institute (2015). Performance Standards for Antimicrobial Susceptibility Testing: Twenty-Fifth Informational Supplement. Wayne, PA: CLSI.

[B8] da Silva N. S. Muniz V. D. Estofolete C. F. Furtado G. H. Rubio F. G. (2014). Identification of temporal clusters and risk factors of bacteremia by nosocomial vancomycin-resistant enterococci. Am. J. Infect. Control 42, 389–392. 10.1016/j.ajic.2013.11.010 24679566

[B9] Dumoulin R. Perez N. C. Gaubert S. Duhutrel P. Brinster S. Torelli R.. (2013). Enterococcal Rgg-like regulator ElrR activates expression of the *elrA* operon. J. Bacteriol. 195, 3073–3083. 10.1128/JB.00121-13 23645602 PMC3697541

[B10] Eckert C. Lecerf M. Dubost L. Arthur M. Mesnage S. (2006). Functional analysis of AtlA, the major *N*-acetylglucosaminidase of *Enterococcus faecalis*. J. Bacteriol. 188, 8513–8519. 10.1128/JB.01145-06 17041059 PMC1698247

[B11] Emirian A. Fromentin S. Eckert C. Chau F. Dubost L. Delepierre M.. (2009). Impact of peptidoglycan *O*-acetulation on autolytic activities of the *Enterococcus faecalis N*-acetylglucosaminidase AtlA and *N*-acetylmuramidase AtlB. FEBS Lett. 583, 3033–3038. 10.1016/j.febslet.2009.08.010 19686739

[B12] Fisher K. Phillips C. (2009). The ecology, epidemiology and virulence of *Enterococcus*. Microbiology 155, 1749–1757. 10.1099/mic.0.026385-0 19383684

[B13] Foo H. Chater M. Maley M. van Hal S. J. (2014). Glycopeptide use is associated with increased mortality in *Enterococcus faecalis* bacteraemia. J. Antimicrob. Chemother. 69, 2252–2257. 10.1093/jac/dku107 24744303

[B14] French G. L. (1998). Enterococci and vancomycin resistance. Clin. Infect. Dis. 27, S75–83. 10.1086/514910 9710674

[B15] Goh E. B. Yim G. Tsui W. McClure J. Surette M. G. Davies J. (2002). Transcriptional modulation of bacterial gene expression by subinhibitory concentrations of antibiotics. Proc. Natl. Acad. Sci. U.S.A. 99, 17025–17030. 10.1073/pnas.252607699 12482953 PMC139263

[B16] Gurung M. Moon D. C. Choi C. W. Lee J. H. Bae Y. C. Kim J.. (2011). *Staphylococcus aureus* produces membrane-derived vesicles that induce host cell death. PLoS ONE 6:e27958. 10.1371/journal.pone.0027958 22114730 PMC3218073

[B17] Heidari H. Emaneini M. Dabiri H. Jabalameli F. (2016). Virulence factors, antimicrobial resistance pattern and molecular analysis of enterococcal strains isolated from burn patients. Microb. Pathog. 90, 93–97. 10.1016/j.micpath.2015.11.017 26620079

[B18] Heidari H. Hasanpour S. Saraie H. S. E. Motamedifar M. (2017). High incidence of virulence factors among clinical *Enterococcus faecalis* isolates in southwestern Iran. Infect. Chemother. 49, 51–56. 10.3947/ic.2017.49.1.51 28332345 PMC5382050

[B19] Hendrickx A. P. van Schaik W. Willems R. J. (2013). The cell wall architecture of *Enterococcus faecium*: from resistance to pathogenesis. Future Microb. 8, 993–1010. 10.2217/fmb.13.66 23902146

[B20] Jeon H. Oh M. H. Jun S. H. Kim S. I. Choi C. W. Kwon H. I.. (2016). Variation among *Staphylococcus aureus* membrane vesicle proteomes affects cytotoxicity of host cells. Microb. Pathog. 93, 185–193. 10.1016/j.micpath.2016.02.014 26924795

[B21] Jun S. H. Lee J. H. Kim B. R. Kim S. I. Park T. I. Lee J. C.. (2013). Acinetobacter baumannii outer membrane vesicles elicit a potent innate immune response via membrane proteins. PLoS ONE 8:e71751. 10.1371/journal.pone.0071751 23977136 PMC3743744

[B22] Jun S. H. Lee J. H. Kim S. I. Choi C. W. Park T. I. Jung H. R.. (2017). *Staphylococcus aureus*-derived membrane vesicles exacerbate skin inflammation in atopic dermatitis. Clin. Exp. Allergy 47, 85–96. 10.1111/cea.12851 27910159

[B23] Kadurugamuwa J. L. Beveridge T. J. (1995). Virulence factors are released from *Pseudomonas aeruginosa* in association with membrane vesicles during normal growth and exposure to gentamicin: a novel mechanism of enzyme secretion. J. Bacteriol. 177, 3998–4008. 10.1128/jb.177.14.3998-4008.1995 7608073 PMC177130

[B24] Kaplan J. B. (2011). Antibiotic-induced biofilm formation. Int. J. Artif. Organs 34, 737–751. 10.5301/ijao.5000027 22094552

[B25] Katsui N. Tsuchido T. Hiramatsu R. Fujikawa S. Takano M. Shibasaki I. (1982). Heat-induced blebbing and vesiculation of the outer membrane of *Escherichia coli*. J. Bacteriol. 151, 1523–1531. 7050091 10.1128/jb.151.3.1523-1531.1982 PMC220434

[B26] Kayaoglu G. Ørstavik D. (2004). Virulence factors of *Enterococcus faecalis*: relationship to endodontic disease. Crit. Rev. Oral Biol. Med. 15, 308–320. 10.1177/154411130401500506 15470268

[B27] Kim S. H. Kim K. H. Kim H. B. Kim N. J. Kim E. C. Oh M. D. (2008). Outcome of vancomycin treatment in patients with methicillin-susceptible *Staphylococcus aureus* bacteraemia. Antimicrob. Agents Chemother. 52, 192–197. 10.1128/AAC.00700-07 17984229 PMC2223910

[B28] Kim Y. J. Jeon H. Na S. H. Kwon H. I. Selasi G. N. Nicholas A.. (2016). *Stenotrophomonas maltophilia* outer membrane vesicles elicit a potent inflammatory response *in vitro* and *in vivo*. Pathog. Dis. 74:ftw104. 10.1093/femspd/ftw104 27756813

[B29] Kuehn M. J. Kesty N. C. (2005). Bacterial outer membrane vesicles and the host-pathogen interaction. Genes Dev. 19, 2645–2655. 10.1101/gad.1299905 16291643

[B30] Kulp A. Kuehn M. J. (2010). Biological functions and biogenesis of secreted bacterial outer membrane vesicles. Annu. Rev. Microbiol. 64, 163–184. 10.1146/annurev.micro.091208.073413 20825345 PMC3525469

[B31] Lee E. Y. Choi D. Y. Kim D. K. Kim J. W. Park J. O. Kim S.. (2009). Gram-positive bacteria produce membrane vesicles: proteomics-based characterization of *Staphylococcus aureus*-derived membrane vesicles. Proteomics 9, 5425–5436. 10.1002/pmic.200900338 19834908

[B32] Lee J. H. Choi C. W. Lee T. Kim S. I. Lee J. C. Shin J. H. (2013). Transcription factor σB plays an important role in the production of extracellular membrane-derived vesicles in *Listeria monocytogenes*. PLoS ONE 8:e73196. 10.1371/journal.pone.0073196 23977379 PMC3748028

[B33] Liu Y. Defourny K. A. Y. Smid E. J. Abee T. (2018). Gram-positive bacterial extracellular vesicles and their impact on health and disease. Front. Microbiol. 9:1502. 10.3389/fmicb.2018.01502 30038605 PMC6046439

[B34] Macdonald I. A. Kuehn M. J. (2013). Stress-induced outer membrane vesicle production by *Pseudomonas aeruginosa*. J. Bacteriol. 195, 2971–2981. 10.1128/JB.02267-12 23625841 PMC3697536

[B35] McBroom A. J. Kuehn M. J. (2007). Release of outer membrane vesicles by gram-negative bacteria is a novel envelope stress response. Mol. Microbiol. 63, 545–558. 10.1111/j.1365-2958.2006.05522.x 17163978 PMC1868505

[B36] Moura T. M. Campos F. S. Caierão J. Franco A. C. Roehe P. M. d'Azevedo P. A.. (2015). Influence of a subinhibitory concentration of vancomycin on the *in vitro* expression of virulence-related genes in the vancomycin-resistant *Enterococcus faecalis*. Rev. Soc. Bras. Med. Trop. 48, 617–621. 10.1590/0037-8682-0017-2015 26516976

[B37] Mug-Opstelten D. Witholt B. (1978). Preferential release of new outer membrane fragments by exponentially growing *Escherichia coli*. Biochim. Biophys. Acta 508, 287–295. 10.1016/0005-2736(78)90331-0 346062

[B38] Nho J. S. Jun S. H. Oh M. H. Park T. I. Choi C. W. Kim S. I. (2015). *Acinetobacter nosocomialis* secretes outer membrane vesicles that induce epithelial cell death and host inflammatory responses. Microb. Pathog. 81, 39–45. 10.1016/j.micpath.2015.03.012 25778390

[B39] Nicholas A. Jeon H. Selasi G. N. Na S. H. Kwon H. I. Kim Y. J.. (2017). *Clostridium difficile*-derived membrane vesicles induce the expression of pro-inflammatory cytokine genes and cytotoxicity in colonic epithelial cells *in vitro*. Microb. Pathog. 107, 6–11. 10.1016/j.micpath.2017.03.006 28284851

[B40] O'Driscoll T. Crank C. W. (2015). Vancomycin-resistant enterococcal infections: epidemiology, clinical manifestations, and optimal management. Infect. Drug Resist. 8, 217–230. 10.2147/IDR.S54125 26244026 PMC4521680

[B41] Pendleton J. N. Gorman S. P. Gilmore B. F. (2013). Clinical relevance of the ESKAPE pathogens. Expert Rev. Anti Infect. Ther. 11, 297–308. 10.1586/eri.13.12 23458769

[B42] Ramos S. Chafsey I. Silva N. Hébraud M. Santos H. Capelo-Martinez J. L.. (2015). Effect of vancomycin on the proteome of the multiresistant *Enterococcus faecium* SU18 strain. J. Proteomics 113, 378–387. 10.1016/j.jprot.2014.10.012 25449832

[B43] Rice L. B. (2008). Federal funding for the study of antimicrobial resistance in nosocomial pathogens: no ESKAPE. J. Infect. Dis. 197, 1079–1081. 10.1086/533452 18419525

[B44] Rivera J. Cordero R. J. Nakouzi A. S. Frases S. Nicola A. Casadevall A. (2010). *Bacillus anthracis* produces membrane-derived vesicles containing biologically active toxins. Proc. Natl. Acad. Sci. U.S.A. 107, 19002–19007. 10.1073/pnas.1008843107 20956325 PMC2973860

[B45] Schweizer M. L. Furuno J. P. Harris A. D. Johnson J. K. Shardell M. D. McGregor J. C.. (2011). Comparative effectiveness of nafcillin or cefazolin versus vancomycin in methicillin-susceptible *Staphylococcus aureus* bacteraemia. BMC Infect. Dis. 11:279. 10.1186/1471-2334-11-279 22011388 PMC3206863

[B46] Shankar N. Baghdayan A. S. Gilmore M. S. (2002). Modulation of virulence within a pathogenicity island in vancomycin-resistant *Enterococcus faecalis*. Nature 417, 746–750. 10.1038/nature00802 12066186

[B47] Shepard B. D. Gilmore M. S. (2002). Antibiotic-resistant enterococci: the mechanisms and dynamics of drug introduction and resistance. Microbes Infect. 4, 215–224. 10.1016/S1286-4579(01)01530-1 11880055

[B48] Sinel C. Cacaci M. Meignen P. Guérin F. Davies B. W. Sanguinetti M.. (2017). Subinhibitory concentrations of ciprofloxacin enhance antimicrobial resistance and pathogenicity of *Enterococcus faecium*. Antimicrob. Agents Chemother. 61, e02763–e02716. 10.1128/AAC.02763-16 28193670 PMC5404537

[B49] Thay B. Wai S. N. Oscarsson J. (2013). *Staphylococcus aureus* α-toxin-dependent induction of host cell death by membrane-derived vesicles. PLoS ONE 8:e54661. 10.1371/journal.pone.0054661 23382935 PMC3561366

[B50] Thompson S. S. Naidu Y. M. Pestka J. J. (1985). Ultrastructural localization of an extracellular protease in *Pseudomonas fragi* by using the peroxidase-antiperoxidase reaction. Appl. Environ. Microbiol. 50, 103810–103842. 3909961 10.1128/aem.50.4.1038-1042.1985 PMC291789

[B51] Toyofuku M. Carcamo-Oyarce G. Yamamoto T. Eisenstein F. Hsiao C. C. Kurosawa M.. (2017). Prophage-triggered membrane vesicle formation through peptidoglycan damage in *Bacillus subtilis*. Nat. Commun. 8:481. 10.1038/s41467-017-00492-w 28883390 PMC5589764

[B52] Wagner T. Joshi B. Janice J. Askarian F. Škalko-Basnet N. Hagestad O. C.. (2018). *Enterococcus faecium* produces membrane vesicles containing virulence factors and antimicrobial resistance related proteins. J. Proteomics 187, 28–38. 10.1016/j.jprot.2018.05.017 29857065

[B53] Wang X. Thompson C. D. Weidenmaier C. Lee J. C. (2018). Release of *Staphylococcus aureus* extracellular vesicles and their application as a vaccine platform. Nat. Commun. 9:1379. 10.1038/s41467-018-03847-z 29643357 PMC5895597

[B54] Yim G. McClure J. Surette M. G. Davies J. E. (2011). Modulation of *Salmonella* gene expression by subinhibitory concentrations of quinolones. J. Antibiot. 64, 73–78. 10.1038/ja.2010.137 21102598

[B55] Yu W. Hallinen K. M. Wood K. B. (2017). Interplay between antibiotic efficacy and drug-induced lysis underlies enhanced biofilm formation at subinhibitory drug concentrations. Antimicrob. Agents Chemother. 62, e01603–e01617. 10.1128/AAC.01603-17 29061740 PMC5740344

[B56] Yun S. H. Park E. C. Lee S. Y. Lee H. Choi C. W. Yi Y. S.. (2018). Antibiotic treatment modulates protein components of cytotoxic outer membrane vesicles of multidrug-resistant clinical strain, *Acinetobacter baumannii* DU202. Clin. Proteomics 15:28. 10.1186/s12014-018-9204-2 30186054 PMC6118003

[B57] Zirakzadeh A. Patel R. (2006). Vancomycin-resistant enterococci: colonization, infection, detection, and treatment. Mayo Clin. Proc. 81, 529–536. 10.4065/81.4.529 16610573

