# Supplementary material for: Production of Membrane Vesicles by Enterococcus faecium Cultured With or Without Subinhibitory Concentrations of Antibiotics and Their Pathological Effects on Epithelial Cells
Source: Front Cell Infect Microbiol. 2019 Aug 14;9:295. doi: 10.3389/fcimb.2019.00295 (PMC6702262; doi:10.3389/fcimb.2019.00295)
Supplement: Supplementary file 1 [file Data_Sheet_1.PDF]

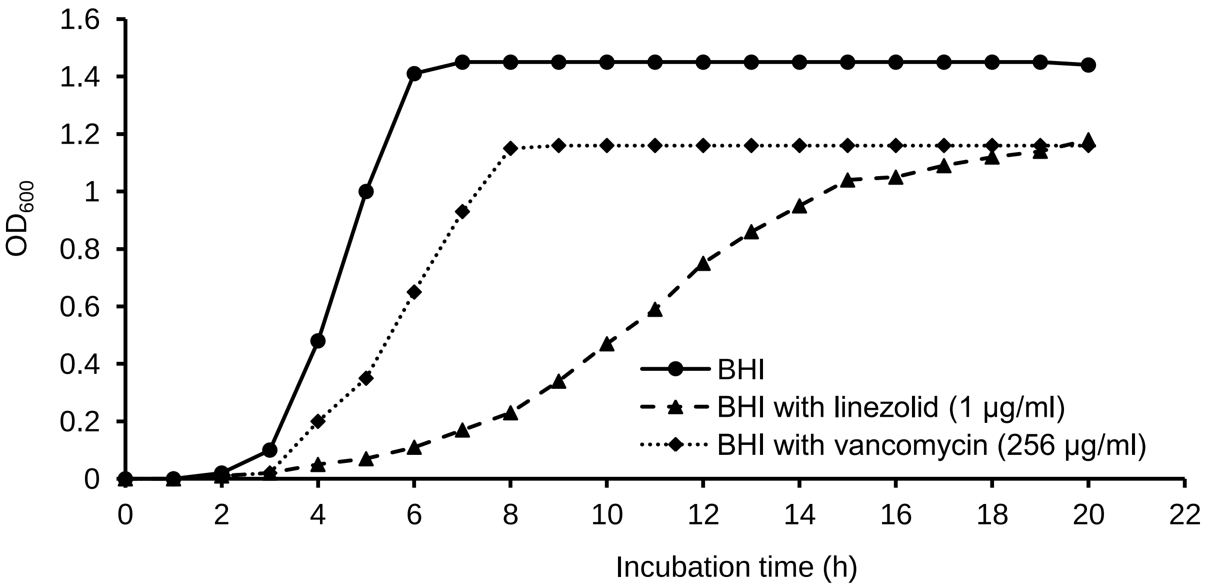


**FIGURE S1.** Growth of *E. faecium* ATCC 700221. Bacteria were cultured in BHI broth, BHI broth with 1 μg/ml linezolid, or BHI broth with 256 μg/ml vancomycin, and bacterial growth was measured using a spectrophotometer at an optical density of A600 ($OD_{600}$) over time. Membrane vesicles were isolated from bacterial cultures at 1.05 at $OD_{600}$.

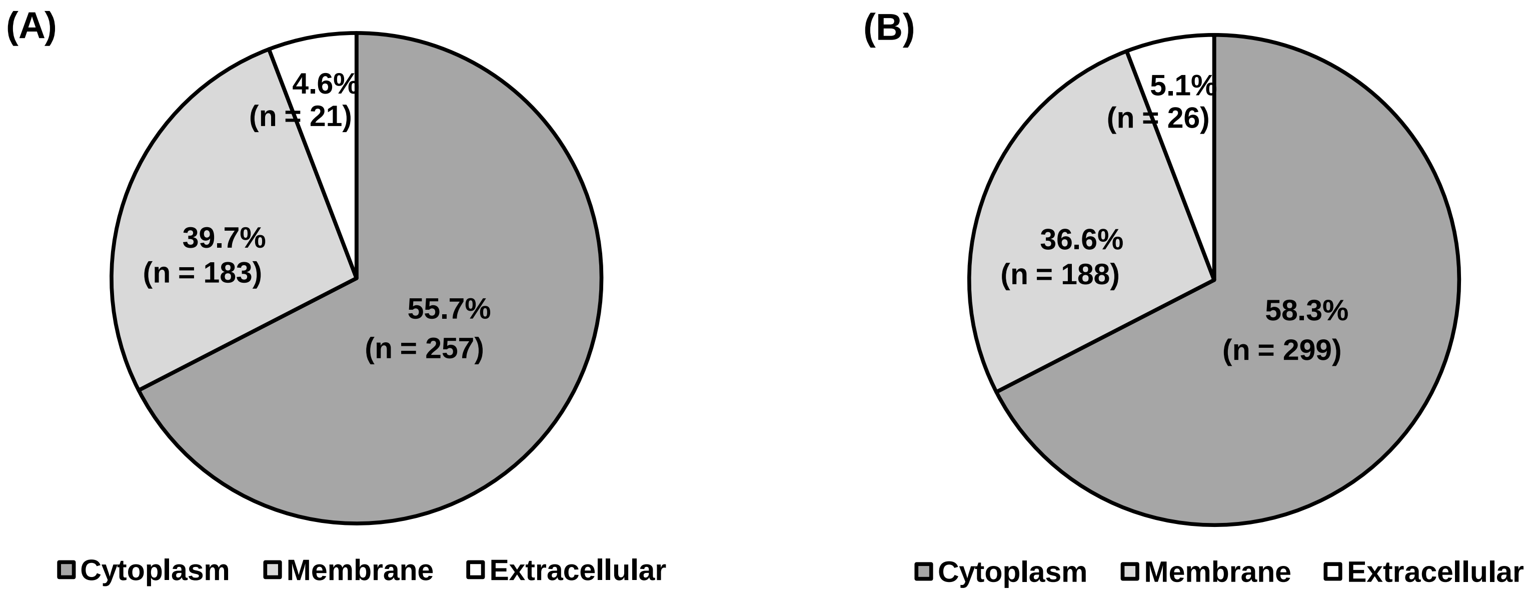


**FIGURE S2.** Cellular localization of MV proteins. MVs were isolated from *E. faecium* ATCC 700221 cultured in BHI broth with 256 µg/ml vancomycin (A) or BHI broth with 1 µg/ml linezolid (B). A total of 461 (A) and 513 (B) proteins were analyzed.

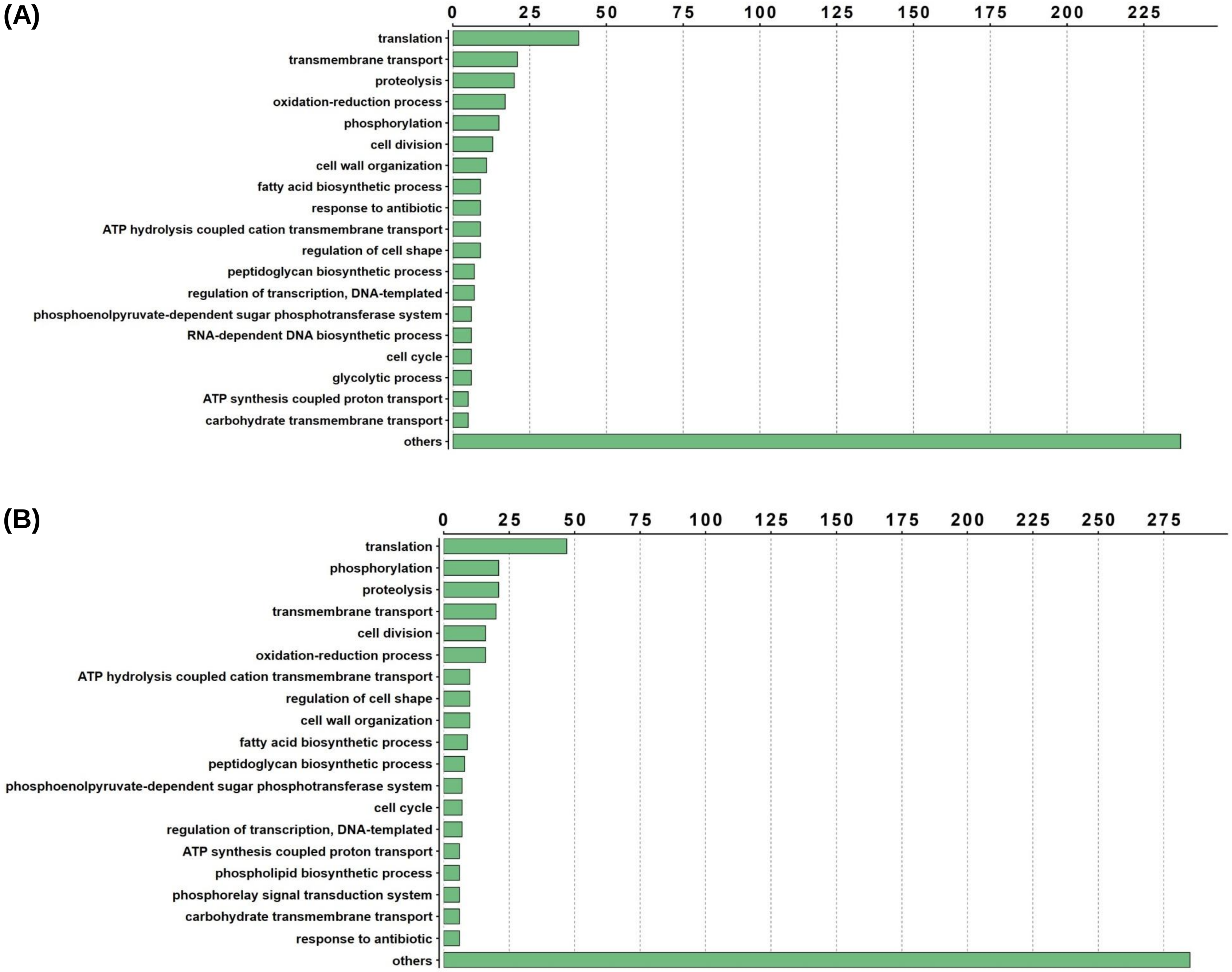


**FIGURE S3.** Classification of MV proteins based on Gene Ontology. MVs were isolated from *E. faecium* ATCC 700221 cultured in BHI broth with 256 μg/ml vancomycin (MVs/VAN) (A) or BHI broth with 1 μg/ml linezolid (MVs/LIN) (B). A total of 461 (A) and 513 (B) proteins in the MVs/VAN and MVs/LIN were analyzed based on Gene Ontology.

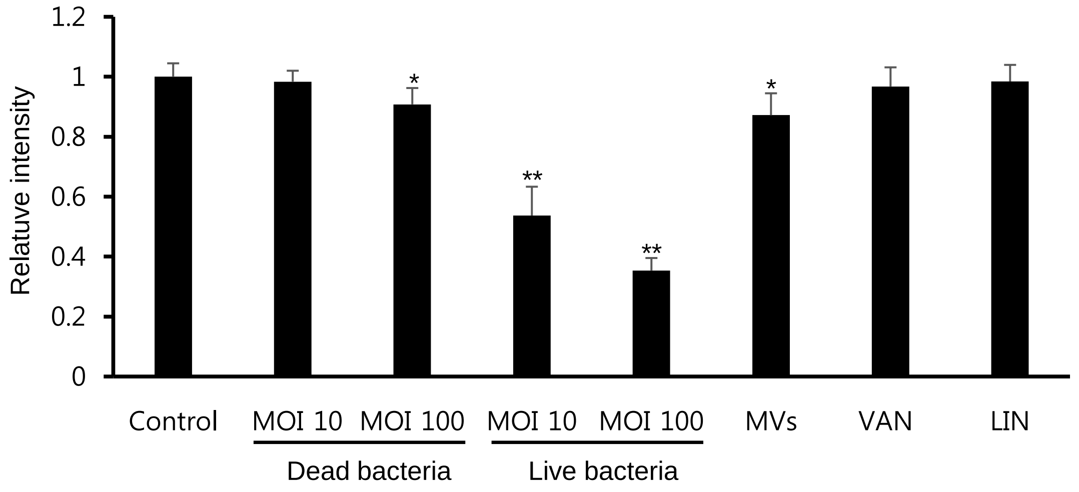


**FIGURE S4.** Cytotoxicity of Caco-2 cells treated with bacteria, MVs, and antibiotics. *E. faecium* ATCC 700221 was cultured in BHI broth to reach 1.05 at $OD_{600}$. Dead bacteria were prepared by fixation with 10% formalin for 20 h and then washing with PBS five times. MVs were isolated from culture supernatants of *E. faecium* cultured in BHI broth. Cells were treated with dead bacteria at multiplicity of infection (MOI) 10 and 100, live bacteria at MOI 10 and 100, MVs (5 μg/ml), vancomycin (256 μg/ml), and linezolid (1 μg/ml) for 24 h. Cell viability was determined using an MTT assay. Data are presented as the mean ± SD of three experiments. * $P < 0.05$, ** $P < 0.01$ compared to untreated control cells.

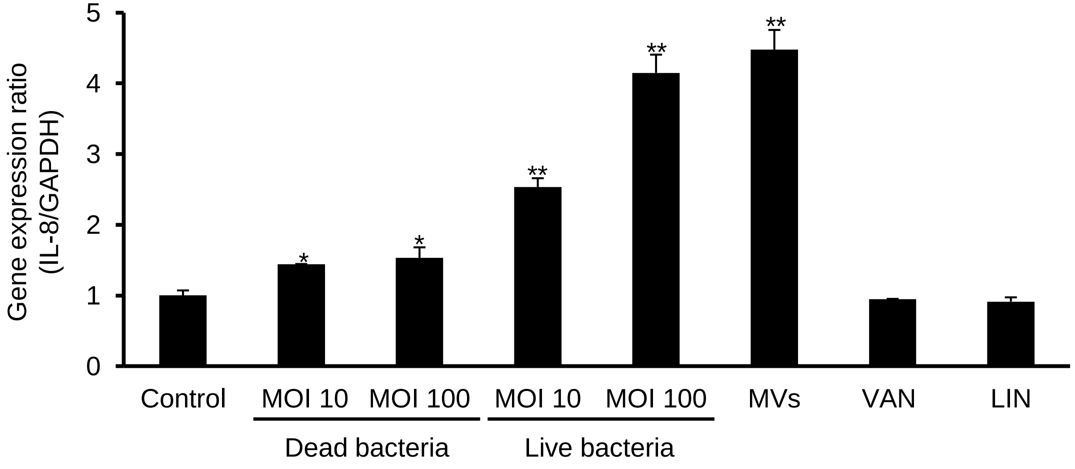


**FIGURE S5.** Expression of *IL-8* gene in Caco-2 cells treated with bacteria, MVs, and antibiotics. *E. faecium* ATCC 700221 was cultured in BHI broth to reach 1.05 at $OD_{600}$. Dead bacteria were prepared by fixation with 10% formalin for 10 min and then washing with PBS three times. MVs were isolated from culture supernatants of *E. faecium* cultured in BHI broth. Cells were treated with dead bacteria at multiplicity of infection (MOI) 10 and 100, live bacteria at MOI 10 and 100, MVs (5 μg/ml), vancomycin (256 μg/ml), and linezolid (1 μg/ml) for 3 h and expression of *IL-8* gene was assessed via qPCR. Data are presented as the mean ± SD of three independent experiments. * $P < 0.05$, ** $P < 0.01$ compared to untreated control cells.

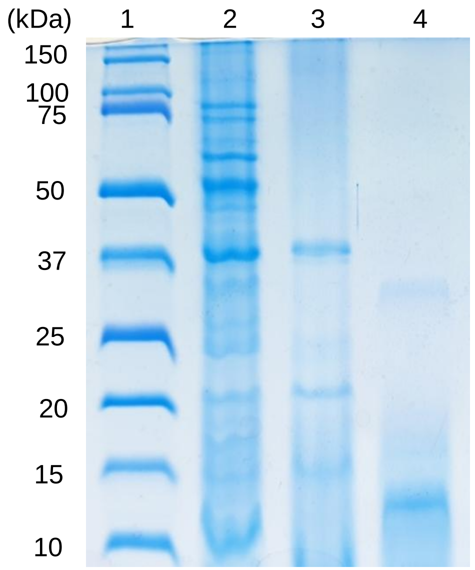


**FIGURE S6** SDS-PAGE analysis of MV proteins. *E. faecium* MVs were treated with 0.1 µg/ml proteinase K for 3 h at 50°C for THE degradation of MV proteins. Lane 1, molecular weight marker; 2, intact MVs/BHI; 3, proteinase K-treated MVs/BHI; 4, proteinase K.
